# Lipid–Protein Interactions in Niemann–Pick Type C Disease: Insights from Molecular Modeling

**DOI:** 10.3390/ijms20030717

**Published:** 2019-02-07

**Authors:** Simon Wheeler, Ralf Schmid, Dan J Sillence

**Affiliations:** 1 School of Pharmacy, De Montfort University, The Gateway, Leicester LE1 9BH, UK; simon.wheeler@dmu.ac.uk; 2 Leicester Institute of Structural and Chemical Biology, Henry Wellcome Building, University of Leicester, Lancaster Road, Leicester LE1 7RH, UK; r.schmid@le.ac.uk; 3 Department of Molecular and Cell Biology, Henry Wellcome Building, University of Leicester, Lancaster Road, Leicester LE1 7RH, UK

**Keywords:** Niemann–Pick, lipids, NPC1, BK, TRPML1, Annexin A2, SNARE

## Abstract

The accumulation of lipids in the late endosomes and lysosomes of Niemann–Pick type C disease (NPCD) cells is a consequence of the dysfunction of one protein (usually NPC1) but induces dysfunction in many proteins. We used molecular docking to propose (a) that NPC1 exports not just cholesterol, but also sphingosine, (b) that the cholesterol sensitivity of big potassium channel (BK) can be traced to a previously unappreciated site on the channel’s voltage sensor, (c) that transient receptor potential mucolipin 1 (TRPML1) inhibition by sphingomyelin is likely an indirect effect, and (d) that phosphoinositides are responsible for both the mislocalization of annexin A2 (AnxA2) and a soluble NSF (N-ethylmaleimide Sensitive Fusion) protein attachment receptor (SNARE) recycling defect. These results are set in the context of existing knowledge of NPCD to sketch an account of the endolysosomal pathology key to this disease.

## 1. Introduction

Niemann–Pick type C disease (NPCD) is a devastating neurological disorder that mostly affects children. Its root cause is dysfunction in the cholesterol-binding proteins NPC1 or NPC2. Low-density lipoprotein (LDL)-derived cholesterol is delivered to the cell in the form of cholesteryl esters. These are carried by the endocytic pathway to the lysosomes where they are hydrolyzed by lysosomal acid lipase (LAL). Unesterified cholesterol (sometimes termed “free cholesterol”) is collected from intraluminal membranes by NPC2 which binds to loop 2 of NPC1 [1,2] and transfers the lipid to the N-terminal domain (NTD) of NPC1 [3,4] (see Figure S1, for the domains of NPC1). This domain then relays the cholesterol to the NPC1 sterol-sensing domain (SSD) [3,5,6] from where the lipid transfers to the endoplasmic reticulum (ER) via a partner protein [7]. The most up-to-date version of this account [2] proposes that cholesterol diffuses from the NPC1 SSD into the luminal side of the lysosomal membrane, spontaneously flips, and is then collected by other organelles. However, cholesterol flipping is rapid [8]; thus, it is unclear why NPC1 deletion would lead to cholesterol accumulation in the lysosome or a cholesterol deficit in the ER, which are in fact cellular hallmarks of NPCD [9,10]. Thus, we asked if there was a cholesterol-binding site on NPC1 aligned with the cytosolic face of the late endosomal/lysosomal (LEL) membrane. If such a site existed, it would explain more fully NPC1′s role in cholesterol export and, thus, the accumulation of cholesterol that results from NPC1′s deletion or mutation.

**Figure 1:**
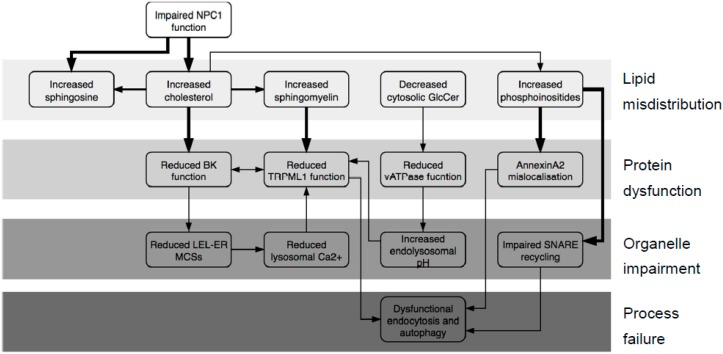
Pathological cascade in Niemann–Pick type C disease (NPCD). All steps are supported by at least one published report referenced in the text; steps shown with bold arrows are the subject of this publication.

This accumulation of cholesterol triggers a secondary build-up of other fats including sphingolipids and phosphoinositides (Figure 1) [11]. This pathology prompted us to postulate that the accumulating lipids could bind to LEL proteins and lead to changes in their function. For example (Figure 1), cholesterol accumulation will likely affect the big potassium channel (BK, also known as Slo1, MaxiK and KCa1.1), which was recently found on lysosomes [12,13]. It is long recognized as being sensitive to its lipid environment, with cholesterol suggested as the key species [14,15,16,17], and there is evidence, in the form of structure–activity studies, to suggest a specific interaction rather than an influence on bilayer thickness [18,19]. Thus, we would expect cholesterol to bind to the BK protein. The lysosomal role of the channel is unclear, though it is probably significant that it acts to regulate Transient receptor potential mucolipin 1 (TRPML1), a Ca^2+^-efflux channel required for membrane fusion and thus endocytosis [12,20]. BK is composed of four identical subunits, each of which contains a voltage sensor and two regulation of conductance for potassium (RCK) domains. Each subunit also contributes two helices to the channel pore (Figure S2). A 2012 paper [21] traced cholesterol sensitivity to a motif (Val509–Lys518) positioned in RCK1; this sensitivity was lost when the protein was cleaved before Val509. This cleavage was quite drastic, removing all of RCK1 and -2, thus significantly weakening the link between Ca^2+^ binding and channel opening, and questioning the physiological relevance of these findings. More importantly still, in the light of more recent structural work on the protein [22,23], it appears that the region identified as binding cholesterol is present in the cytosol where levels of cholesterol will be lower than in the membrane. Given that the membrane region has four cholesterol-binding motifs (Figure S2), this seems questionable. Thus, we wished to revisit the question of where cholesterol binds to BK.

The accumulation of sphingomyelin (SM) in the LELs of NPCD cells is triggered by increased levels of cholesterol. SM was reported to inhibit Ca^2+^ efflux through the TRPML1 channel [24] (Figure 1), something that is necessary for the correct completion of endocytosis [20]. SM accumulation, therefore, will contribute to endocytic failure; thus, we wished to identify where SM binds to TRPML1. A recent structural study [25] revealed that increased endolysosomal pH also compromises TRPML1 function. This channel exists as a homotetramer whose assembly is driven by interactions between the loop 1 regions on adjacent subunits (Figure S3). An aspartate-rich region on each loop, thus, forms a pore which confers pH-sensitivity on the channel. At low pH, the aspartate residues are all protonated; thus, Ca^2+^ ions can pass freely through. As pH rises, the aspartates become deprotonated, and the resulting anionic carboxylate moieties bind Ca^2+^, thereby inhibiting its own conductance. In agreement with previous reports [26,27], we have unpublished data [28] that the pH of the LEL compartment in NPCD is increased, and that the increase stems from defective activation of the vATPase proton pump by glucosylceramide (Figure 1). This is another reason for expecting reduced TRPML1 function in NPCD.

Whilst the assembly of cholesterol and sphingolipids in ordered domains termed lipid rafts is widely appreciated, it is less well known that cholesterol also mediates clustering of phosphoinositides (Figure 1, reviewed in References [29,30]). Therefore, it seems likely that cholesterol accumulation in NPCD will lead to trapping of phosphoinositides in LEL vesicles; indeed, phosphoinositides do increase in the LEL compartment of NPCD cells [11]. These negatively charged lipids are likely to bind to particular proteins; thus, the accumulation of phosphoinositides may lead to the accumulation of such proteins and, thus, impaired protein function.

It is known for some time that the peripheral membrane protein annexin A2 (AnxA2) mislocalizes in NPCD, associating with late, not early, endosomes [31,32]. Multiple studies reported the association of AnxA2 with lipid rafts [33,34,35], although phosphatidylinositol-4,5-bisphosphate (PI(4,5)P_2_) was also reported as the key binding lipid [36,37]. The clustering of phosphoinositides by cholesterol offers a way of unifying these disparate observations. Therefore, we asked if phosphoinositides could bind to AnxA2 (Figure 1).

Endocytosis and autophagy, both of which are dysfunctional in NPCD [38,39], rely on membrane fusion events, which in turn are dependent on soluble NSF (N-ethylmaleimide Sensitive Fusion) protein attachment receptor (SNARE) proteins such as syntaxins and vesicle-associated membrane proteins (VAMPs). The subsequent re-use of these proteins, therefore, requires their release from the assembled SNARE complex, and this process is retarded in cholesterol-rich membranes [40]. Significant work was done on the part of syntaxin 1 (Stx1) adjacent to the membrane (the juxta-membrane region) which is polybasic and thus polycationic in vivo [41,42,43,44]. Many other syntaxins have a similar region, as do their VAMP partners (see multi-sequence alignments, Figure S4). Thus, we wished to examine the question of whether phosphoinositides, not cholesterol, are directly responsible for retarded SNARE recycling (Figure 1).

To examine lipid–protein interactions, it is necessary to identify the region of the protein where the lipid associates or binds. A protein’s sequence can be searched for established lipid-binding motifs using computer algorithms (e.g., FuzzPro, bioinformatics.nl/cgi-bin/emboss/fuzzpro), but the predictive power of this technique is variable. For instance, the most common cholesterol-binding motif, termed CRAC [45,46] (cholesterol recognition amino-acid consensus) is defined as L/V-X_1-5_-Y-X_1-5_-R/K and, thus, is of variable length and strictly defined at only one position. Thus, algorithmic searching for this motif typically generates numerous false positives for any given protein sequence. The same is true of the reverse motif CARC [47], which also binds cholesterol. In contrast, a sphingolipid-binding pattern [48] was postulated as a post hoc rationalization of experimental observations in a small number of proteins, though this was not extrapolated to a general motif. More recently, the protein p24 was found to bind to sphingomyelin via a defined region [49]. Based on this finding, a set of sphingolipid-binding motifs, e.g., VX_2_V_2_X_2_LF, was elucidated [50]. However, to our knowledge, no interactions between sphingolipids and specific residues within these motifs were identified, nor is it clear where the required terminal aromatic residue of the motif should be positioned relative to the membrane in which the protein is embedded. Thus, algorithmic searching of sequences for binding motifs offers only an approximate guide to where lipid–protein interactions may occur, and gives only limited information of three-dimensional (3D) features of such interactions. We set out to use molecular docking, which not only allows the exploration and rationalization of lipid binding in structural detail, but also allows ruling out some sites if binding proves impossible or implausible. This technique is, hence, much more reliable than merely identifying motifs.

Such docking requires a structural model of the protein in question, which can be gained by experiment (X-ray, cryoEM, or NMR) or derived from such a structure (by homology modeling or threading). Lipid binding to specific sties of the protein can then be estimated with the protein mostly rigid, but giving flexibility to some designated side chains, while the lipid is allowed to vary its position, orientation, and bond angles. We started our calculations with the bond angles in an energy-minimized state; the docking software randomly selects a starting position and orientation. All these parameters are then systematically altered until an energetically favorable binding “pose”—assessed by an energy scoring function—is discovered, if possible. This procedure is repeated multiple times. and the results are ranked to show more or less energetically favorable binding poses. These poses are grouped into clusters defined by root-mean-square (RMS) difference of atomic positions with the cut-off for membership of a cluster set at >2 Å. The calculations examine only a sample of all possible combinations of parameters and are limited by the accuracy of the scoring function. Thus, the results are hypotheses that need to be inspected and tested—a binding pose with a favorable energy score might be biologically impossible.

**Figure 2:**
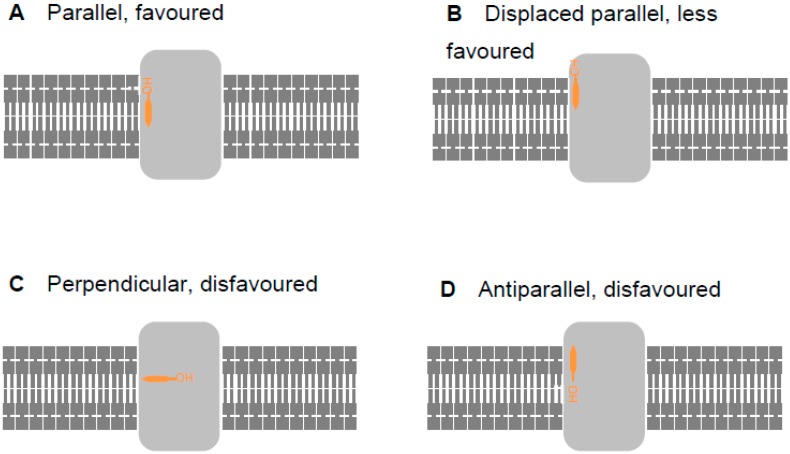
Four possibilities for cholesterol binding to a trans-membrane protein displayed in cartoon form. Trans-membrane proteins (light grey) are embedded in the lipid bilayer (dark grey). Cholesterol (orange) may bind parallel to the membrane (**A**, energetically favored), parallel to the membrane but vertically displaced from it (**B**, energetically less favored), perpendicular to the membrane (**C**, energetically disfavored), or antiparallel (**D**, energetically disfavored).

With membrane-bound and -associated proteins, such as those considered here, the orientation of the protein in the membrane (assessed here using the Orientation of Proteins in Membranes (OPM) database, opm.phar.umich.edu/ [51]) offers a significant clue as to the biological plausibility of binding poses. Thus, a lipid, illustrated by cholesterol (orange) in Figure 2, may bind to a trans-membrane protein (light grey) parallel to the lipophilic section of the membrane and with the hydroxy group aligned with other lipid head groups (Figure 2A). This is the most energetically favorable situation. However, the lipid may also be parallel to the membrane, but vertically displaced from it (Figure 2B). Whilst possible [52], this requires a deformation in the membrane which attracts an energy penalty; such a pose would, therefore, be energetically less favored. Burying the hydroxy group inside the lipophilic portion of the membrane by orienting it in a perpendicular (Figure 2C) or antiparallel (Figure 2D) fashion is still possible, but energetically disfavored. In this work, we considered binding poses similar to those in Figure 2B–D only when calculations did not generate poses corresponding to the more favorable situation shown in Figure 2A.

We made our calculations more computationally tractable by neglecting the membrane; thus, we assessed only lipid–protein interactions. Lipid–protein interactions considered in isolation may be energetically favorable with the lipid in perpendicular or antiparallel situations. As the lipid starting position and orientation are randomized, some of the iterations of the docking calculations will start with the lipid in a position perpendicular or antiparallel to the lipophilic section of the membrane. Thus, some docking runs will start with the lipid in an orientation that is energetically favorable when considered in isolation but energetically disfavorable when considered in the context of the membrane as a whole. Such an orientation is biologically implausible. Energetic refinement of this orientation will not necessarily result in the lipid adopting a parallel orientation. Therefore, some docking runs may result in binding poses that appear energetically favorable because they only consider lipid–protein interactions but would be energetically disfavorable overall and, thus, biologically implausible. This is a consequence of the randomization of the initial position of the lipid, and not a flaw in the docking experiments.

Whilst this discussion applies mostly to cholesterol, similar considerations pertain to other lipids. Aliphatic lipid tails generally make non-specific interactions with trans-membrane proteins; thus, we routinely perform docking calculations with only the lipid head group to focus attention on the distinctive part of the molecule. These head groups include the first carbon atoms of the aliphatic chains, and it is quite possible that the randomized initial positioning for the docking calculation will orient these atoms and, thus, the alkyl chains, away from the membrane and toward the cytosol. It is also possible that the positioning will point the alkyl chains in opposite directions, one toward the membrane and one toward solvent (referred to as a splayed arrangement in Table S1). It is equally possible that the energy minimization, because done in isolation from the membrane and solvent, will retain either of these orientations if adopted by the initial random positioning. Thus, once again, docking calculations may output binding poses that are energetically favorable in isolation but disfavorable overall and, thus, biologically implausible. This does not indicate a flawed method.

For this reason, it is necessary that the output of such docking be firstly inspected for biological plausibility. Our figures show representative binding poses that result from the docking runs—these are both energetically favorable and biologically plausible. Table S1 presents our extended data including number of clusters, cluster size, assessment of favorability, and interacting residues. Once filtered for energetic favorability and biological plausibility, the results of docking experiments should next be tested by “wet” experiments. Whilst we present no new lab data here, our results are fully consistent with existing findings from other labs which are cited extensively in the text. The main proposal of this work is a set of lipid–protein interactions that explain and connect disparate data from the literature and are testable in vitro. We propose that errors in lipid distribution in NPCD are the proximate cause of protein dysfunction, and that this failure leads eventually to defects at the cellular level. We depict this in graphical form in Figure 1.

## 2. Results

### 2.1. What Does the NPC System Do?

**Figure 3:**
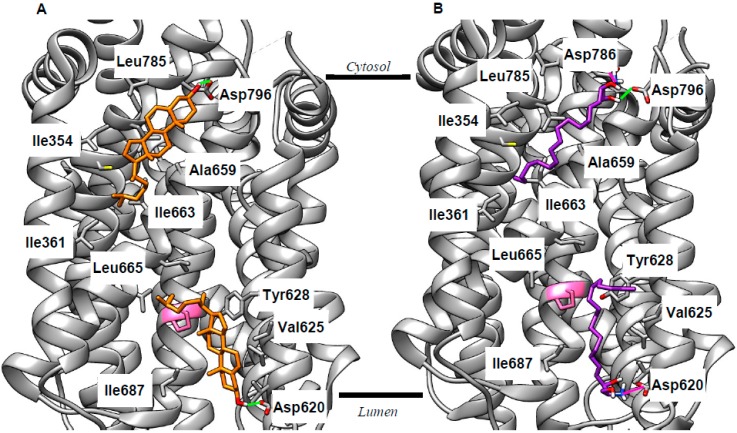
Docking identifies two putative binding pockets on NPC1 aligned to each side of the lysosomal membrane. Sample poses for both cholesterol (orange, **A**) and sphingosine (purple, **B**) show binding in these pockets consistent with evidence of NPC1’s role as an exporter of both lipids. Selected interacting residues are labeled; H-bonds are shown in green (Sph and cholesterol), and ionic interactions are shown in magenta (Sph only). Pro691 is shown in pink; mutation of this residue results in reduced cholesterol binding [53,54]. Lines indicate the approximate position of the membrane (derived from the Orientation of Proteins in Membranes OPM database [51]).

As discussed above, the current view of cholesterol export via NPC1 [2] does not fully explain the lipid misdistribution resulting from mutation or deletion of this protein. This cholesterol export narrative can, however, be made consistent with current knowledge if it is refined to include a binding site on the cytosolic face of the LEL membrane which delivers cholesterol to the ER. Thus, we performed cholesterol docking to the entire SSD of NPC1 using the recently published X-ray structure (Protein Data Bank (PDB) 5U74 [6]). We found lipid-binding poses in two energetically favorable clusters aligned to both the luminal and cytosolic faces of the membrane. Representative examples are shown in Figure 3A; extended results are given in Table S1. In both cases, binding is anchored by an H-bond between the cholesterol hydroxy group and the side chain of an Asp residue, whilst the remainder of the sterol is encased in a cavity formed from lipophilic side chains (Figure 3A; Table S1). These side chains make van der Waals contact with the cholesterol molecule. Crucially, Pro691 is among the residues predicted to interact with cholesterol (pink in Figure 3). Mutation of this residue was shown to reduce cholesterol export [53] and to reduce labeling of NPC1 by a photoactivatable cholesterol analog [54], implying that Pro691 plays an important role in cholesterol binding. The emergence of this residue from docking calculations forms an important agreement between in vitro and in silico approaches. The suggestion of two binding pockets means that NPC1 can potentially be added to the list of proteins featuring cholesterol binding in both leaflets of a lipid bilayer [55,56], a phenomenon termed the “mirror code” [57]. The path between these two binding sites remains to be uncovered, but we propose that NPC1 interacts with the ER to deliver cholesterol from the flipped pocket. This modified narrative takes account of the rapid flipping of cholesterol [8], and the cholesterol deficit at the ER [10] in NPCD, and is, thus, more consistent with available data than that proposed previously [2].

Experiments with lipid profiling and photoactivatable probes demonstrated increased levels of sphingosine (Sph) in NPCD, and that this accumulation can be traced to the LEL [58,59,60], supporting the idea that NPC1 can act as a sphingosine export protein. To investigate this further, we performed our docking experiments with Sph and the SSD of NPC1. Consistent with the studies just cited, we found plausible binding poses for sphingosine aligned to both sides of the LEL membrane. Reminiscent of cholesterol, the polar head group of Sph forms H-bonds or ionic interactions with the side chains of Asp residues, while the lipophilic tail makes van der Waals contact with the same set of lipophilic side chains as cholesterol (Figure 3B; Table S1). We also docked Sph to NPC2 and the NTD of NPC1 (Figure S5). Our calculations gave energetically favorable binding of Sph with the polar head group oriented the same way as cholesterol. This is known to be critical for the correct functioning of the cholesterol export machinery [3,5] and, thus, further supports the idea that Sph may use the same pathway. The consequences of impaired Sph export for LEL function are unclear, though it is perhaps significant that this lipid was recently reported as an inhibitor of glucosylceramidase 2 [61], a target of miglustat [62], which is the only clinically used treatment of NPCD. Alternatively, sphingosine (pKa 6.6 [63]) is likely to be protonated in the endolysosome (pH 4–5) but deprotonated in the cytosol (pH 7.4) and, thus, sphingosine export may act to move H^+^ ions from one to the other. NPC1 deficiency will, therefore, lead to changes in pH in both the LEL and cytosol. This hypothesis could be tested by looking for changes in pH homeostasis in both compartments.

### 2.2. Revising the BK–Cholesterol Interaction Site

**Figure 4:**
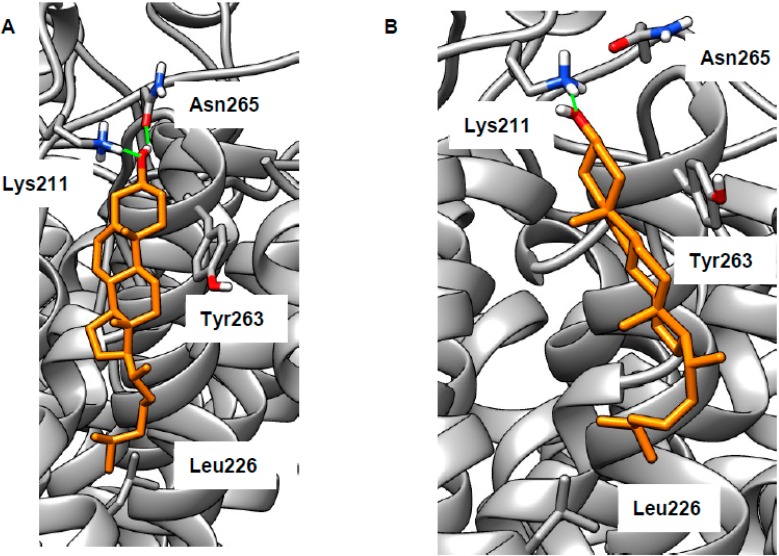
Sample cholesterol-binding poses at the putative binding site on the voltage sensor of big potassium channel (BK). Cholesterol (orange) is able to form H-bonds (green) with Lys211 (**A**,**B**) and Asn265 (**A**), a CH–π interaction with Tyr263 (**B**), and a hydrophobic interaction with Leu226 (**A**,**B**).

As stated in the introduction, we wished to reconsider cholesterol binding to BK in the membrane region. To do this, we built a model of the membrane section of the human BK channel based on a recent cryoEM structure (PDB 5TJI) [22,23]. We attempted docking of cholesterol at each of each of the intra-membrane cholesterol binding motifs. For CARC, CRAC1, or CRAC3 motifs (defined as in [21]), no poses could be found that were both energetically favorable and biologically plausible (assessed as described in the introduction). We then attempted to dock cholesterol in the space near the CRAC2 cholesterol-binding motif (Val258–Arg266, located on the voltage sensor), allowing various combinations of side chains to be flexible. Using this procedure, we found a number of plausible binding poses (representative examples are shown in Figure 4; extended results are given in Table S1). The cholesterol hydroxy group can form H-bonds with Lys211 and/or Asn265 (in some poses, these residues also H-bonded to each other, data not shown). The lipophilic portion of cholesterol makes van der Waals contacts with various lipophilic side chains (Table S1), in particular Leu226, which has favorable interactions with the terminus of cholesterol. In some poses, the side chain of Tyr263 makes CH–π interactions with the smooth α-face of cholesterol (Figure 4B). Thus, these poses feature the classical elements of a CRAC motif (basic, aromatic, and lipophilic residues) [46], but are not derived from a contiguous section of protein sequence, something previously observed in the β2 adrenoceptor [64]. Whilst our results cannot disprove cholesterol binding in the cytosolic domains, we believe that cholesterol binding in the membrane region is more likely. Our docking is fully consistent with previous studies demonstrating BK’s cholesterol sensitivity [14,15,16,17,18]. In particular, both of the representative poses shown in Figure 4 rely on the correct cholesterol stereochemistry, which was shown to be key to its effects on the BK channel [18,19]. Our hypothesis could be tested by mutagenesis experiments involving key hydrogen-bonding residues Lys211 and Asn265.

The implications of cholesterol-induced BK dysfunction for NPCD are multiple (Figure 1). Firstly, this channel as reported as a regulator of LEL–ER membrane contact sites (MCSs) and, thus, to be important for refilling lysosomal Ca^2+^ stores [13] which are reported deficient in NPCD [58,65]. It is also significant that blocking lysosomal Ca^2+^ refilling gave a cellelar phenotype resembling that of lysosomal storage disorders LSDs [66], a class of which NPCD is a member. BK was also reported as mutually regulating lysosomal Ca^2+^ channel TRPML1 [12] (see next section). Whatever the details, the importance of BK for NPCD is attested by the repair of the endocytic defect by either channel agonism [67] or over-expression [12].

### 2.3. LEL Ca^2+^ and TRPML1

**Figure 5:**
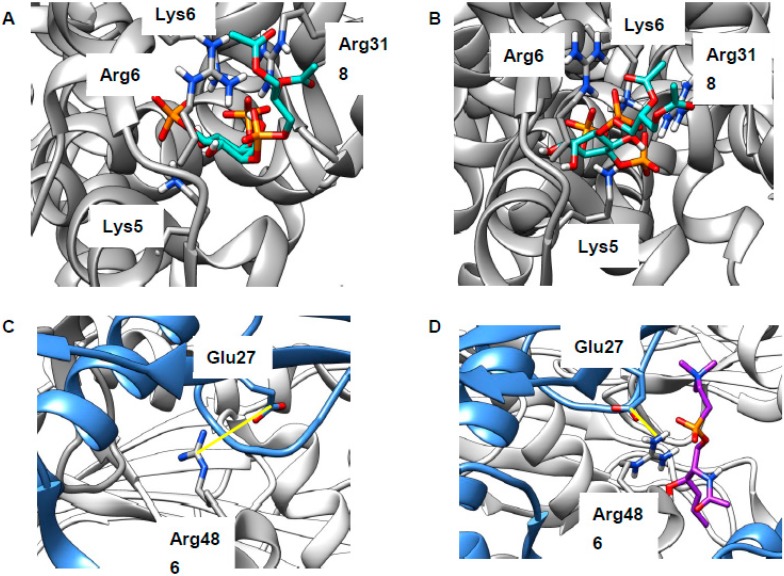
Putative binding sites of lipids at Transient receptor potential mucolipin 1 (TRPML1). (**A**,**B**) Modeling supports mutagenesis experiments identifying a polybasic region as key the binding site for phosphatidylinositol-3,5-bisphosphate (PI(3,5)P_2_) (green, **A**) and PI(4,5)P_2_ (green, **B**). (**C**) Glu276 from one chain (blue) forms a charged interaction with Arg486 from another (grey); interatomic distance: 3.77 Å. (**D**) Modeling suggests plausible binding poses for SM (purple, typical example shown), but these do not disturb the inter-residue interactions present in the apo state; interatomic distance: 2.97 Å. Protein oriented as in Figure S3; distance markers are shown in yellow.

Impaired formation of LEL–ER contact sites is likely to result in reduced concentration of lysosomal Ca^2+^, which was indeed found in NPCD [58,65], though not without some disagreement [24]. Reduced LEL Ca^2+^ will in turn result in impaired calcium release via the TRPML1 channel (Figure 1), something that is necessary for the correct completion of endocytosis [20]. As discussed above, sphingomyelin (SM) accumulates in the LELs of NPCD cells and is reported to inhibit conductance through this channel [24]. There are at least three possibilities for the site of this interaction: (1) SM binds at the same site as synthetic ligand ML-SA1, (2) SM competes with the natural ligand PI(3,5)P_2_, or (3) SM inhibits assembly of the four proteins into the channel (Figure S3A). The first of these is precluded, as the ML-SA1 binding site lies in the intra-membrane region [68,69] and will, thus, be inaccessible to the SM head group. The second was investigated by docking the relevant lipid head groups to a region of the human protein (PDB 5WJ9, [68]) recently identified in a marmoset TRPML3 channel [70] (Figure S3C). Use of just the head groups focused attention on the distinctive part of the lipid (hydrophobic tails are common to most classes), as well as rendering the calculation more tractable. Both PI(3,5)P_2_ and natural inhibitor PI(4,5)P_2_ were found to bind to this polybasic region, thus supporting mutagenesis experiments [70] locating binding here (poses not shown, extended data in Table S1). SM was also successfully docked to this region (poses not shown; extended data in Table S1) but the energy of interaction was much weaker, suggesting that SM will not compete with phosphoinositides for this binding site. Inhibiting channel assembly (the third possibility) was previously shown to lead to reduced TRPML1 function [25] and two sites of inter-chain binding were identified in structural studies: Arg146–Val175 (H-bonded) in the linker region [25] and Arg486–Glu276 (zwitterionic) in the juxta-membrane region [68] (Figure S3A). The Arg146–Val175 site is too far from the membrane to be relevant (Figure S3). We carried out docking of the SM head group to the Arg486–Glu276 region. Very few energetically favorable binding poses positioned the SM head group in a way that would align correctly with the LEL membrane. Those that did feature interactions between the lipid and the protein, but also the protein side chains, preserve interactions between them rather than become separated by SM. Channel assembly is, thus, maintained rather than disrupted in the presence of this polar lipid (Figure 5C,D). Considering this, direct effects of SM on the TRPML1 channel appear unlikely and we believe that the inhibitory properties of this lipid may well derive from indirect effects, perhaps mediated through membrane organization.

TRPML1 plays roles in endocytosis [20,71] and autophagy [72]; thus, the failure of these processes in NPCD [38,39,73,74] is unsurprising (Figure 1), although there may be some redundancy with the two-pore channels (TPCs) [75,76], P2X_4_ [77] and P/Q-type voltage-gated calcium channel [78,79].

### 2.4. Cholesterol Clusters Phosphoinositides—Implications for Annexin Localization

In the introduction, we raised the possibility that cholesterol-mediated clustering of phosphoinositides [29,30] might be invoked to explain the mislocalization of Annexin A2 (AnxA2) in NPCD [31,32]. AnxA2 is a peripheral membrane protein, suggesting that it is likely to associate with membranes via interactions between surface residues with side chains that extend from the protein and head groups that protrude from the membrane. Indeed, previous site-directed mutagenesis identified Lys279 and Lys281 as important for PI(4,5)P_2_ binding [36].

**Figure 6:**
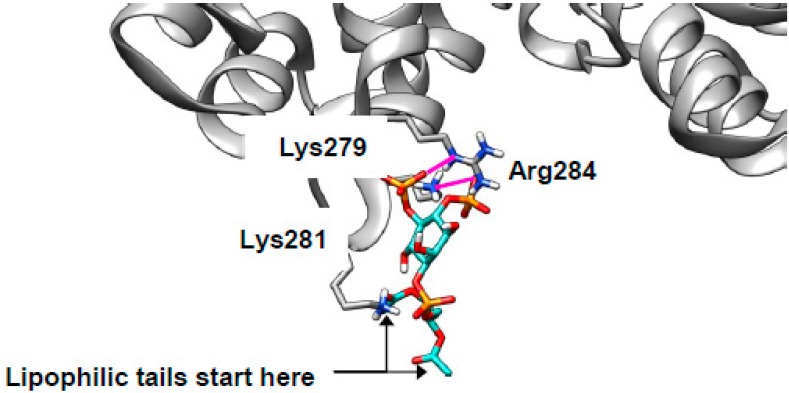
PI(4,5)P_2_ can bind to annexin A2 (AnxA2). PI(4,5)P_2_ (turquoise) can bind to the convex face of AnxA2 (grey) driven by zwitterionic interactions (magenta) with Lys279, Lys281, and Arg284.

With this in mind, we attempted to dock AnxA2 (PDB 2HYW [80]) with PI(4,5)P_2_ using just the lipid head group for the reasons discussed above. The search was concentrated in a space including Lys279, Lys281, and a nearby basic residue Arg284. These residues are expected to be protonated and, thus, cationic in vivo. Consistent with the previously reported results [36,37], our docking experiments show these cationic residues making zwitterionic interactions with the two anionic phosphate groups from the lipids (example binding pose shown in Figure 6, ionic interactions in magenta; extended data shown in Table S1). The two lipid side chains point away from the protein and remain in the membrane. The start of the lipophilic tails is indicated in Figure 6 (one is obscured behind Lys281). Docking of SM was also performed, and the results were much less energetically favorable than for PI(4,5)P_2_ (Table S1) suggesting that the phosphoinositide is genuinely responsible for AnxA2 binding. Thus, in NPCD, AnxA2 will be expected to mislocalize away from early endosomes and to cholesterol-rich late endosomes as observed [31,32] and contribute to the documented endocytic defect [38,73]

### 2.5. Cholesterol Clusters Phosphoinositides—Implications for SNARE Complex Disassembly

**Figure 7:**
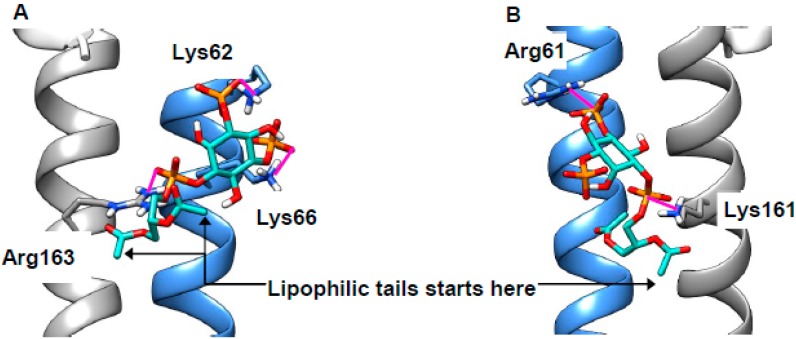
PI(4,5)P_2_ can bind to two members of a SNARE bundle simultaneously. Sample poses of PI(3,5)P_2_ (turquoise) bridging vesicle-associated membrane protein 8( VAMP8) (blue) and syntaxin 7 (grey) in the assembled Soluble NSF (N-ethylmaleimide Sensitive Fusion) protein attachment receptor (SNARE) bundle with zwitterionic interactions (magenta) (**A**,**B**).

In the introduction, we noted that SNARE recycling from cholesterol-rich membranes is retarded [40]. It was postulated that the cholesterol mediates the clustering of phosphoinositides [29,30] and that phosphoinositides might bind to SNARE components and, thus, trap them in membranes. To test this idea, we built a model of a SNARE complex featuring Stx7 and VAMP8—a combination reported as necessary for endocytosis [81]—based on an X-ray structure (PDB 3HD7 [82]) of a related bundle. The juxta-membrane basic residues (VAMP8: Arg67, Lys68, Lys72; Syntaxin 7: Arg232, Lys233, Arg235), as with AnxA2, will be expected to be protonated and, thus, cationic. These extend from the protein in different directions, and it was, therefore, necessary to select appropriate combinations of these to be flexible in the docking experiments. When we attempted docking of PI(3,5)P_2_ to this protein complex, we found numerous plausible poses. As expected, the anionic phosphates on the lipid form zwitterionic interactions with the cationic residues on the protein (magenta, Figure 7A,B; extended data in Table S1). The first carbon atoms of the lipophilic tails are indicated and are oriented toward the membrane (Figure 7A,B). Importantly, the phosphates interact simultaneously with residues on both SNARE partners. This would be expected to promote SNARE interaction and, thus, inhibit complex disassembly. Our results are, therefore, consistent with both the cholesterol-mediated clustering of PI(3,5)P_2_ [29,30] and the recycling defect observed in cholesterol-rich membranes [40], and they add a previously unappreciated subtlety to dysfunctional endocytosis in NPCD. Repeated attempts to dock PI(4,5)P_2_ to our model consistently failed to give plausible binding poses, suggesting that clustering is dependent on only the 3,5-isomer. The situation with autophagy is more complicated, as this was reported to rely on Stx17 in both mammals and *Drosophila* [83,84]; Stx7 does not appear to be involved [85]. Stx17 is unusual in its possession of two trans-membrane helices, neither of which has a polybasic juxta-membrane region (Figure S4).

The widespread effects of phosphoinositides on ion channels [86], not least as agonists of TRPML1 [87,88] and the TPCs [89], suggest that cholesterol-mediated clustering of these lipids will be a fruitful area for future research in lysosomal storage disorders.

## 3. Discussion

We argued here that the accumulation of cholesterol in the LEL compartment results from dysfunctional NPC1 and affects some proteins directly (e.g., BK). At the same time, it triggers the secondary build-up of other lipids which affect other proteins (e.g., AnxA2). Thus, the misdistribution of lipids in the LEL membrane results in widespread protein dysfunction. Ultimately, these defects manifest as errors at the cellular level (Figure 1). The account presented here concerns proteins of the LEL membrane and, even on that restricted basis, is a simplification as, to say nothing of other organelles and processes, there is evidence that AnxA6 [90], CLC-6 [26,79], mTOR [55], rab9 [91,92], and TPC1 [60] are also involved. The absence of high-quality structural data and/or inconclusive in vitro results meant a modeling approach was inappropriate for these proteins at this time.

## 4. Materials and Methods

Multi-sequence alignments were performed in Clustal Omega (ebi.ac.uk/Tools/msa/clustalo/) [93] and visualized in JalView 2.10.5 (http://www.jalview.org/) (version, manufacture, city, if any state, country) [94]. Lipid-binding motifs were located using Fuzzpro (bioinformatics.nl/cgi-bin/emboss/fuzzpro).

Protein structures were either downloaded from the PDB (5U74 for NPC1, 5WJ9 for TRPML1, 2HYW for AnxA2) or models were built using SwissModel (https://swissmodel.expasy.org/interactive) (version, manufacture, city, if any state, country) [95] (5TJI as a template for BK, 3HDY as a template for SNARE bundle). Quality was assessed using QMEANBrane (https://swissmodel.expasy.org/qmean/) (version, manufacture, city, if any state, country) [96,97]; for details, see the Figure S5. Approximate positions in the membrane were found using the OPM database (http://opm.phar.umich.edu/) [51]. The AnxA2 structure features calcium ions which by default are set to zero charge by AutoDock. Thus, atomic charges for these and spatially proximate atoms were calculated using the Atomic Charge Calculator (webchem.ncbr.muni.cz/Platform/ChargeCalculator) [98], and the relevant AutoDock files were manually edited. Ligand structures were prepared in Avogadro (avogadro.cc/) and minimized using the MMF94 force field with at least 5000 steps; other settings were defaults. The lipids considered are shown in Figure S6.

Docking of lipids to proteins was performed using AutoDock 4.2.6 (http://autodock.scripps.edu/) (version, manufacture, city, if any state, country) [99,100] using default settings. Search spaces, the residues allowed to be flexible, and the number of algorithm runs are given in Figure S7. AutoDock clusters binding poses by RMS distance (cut-off >2 Å). Docking scores were used as a preliminary assessment, followed by manual inspection for biological plausibility as discussed in the introduction.

Protein structures, including docking results, were visualized in UCSF Chimera (https://www.cgl.ucsf.edu/chimera/) (version, manufacture, city, if any state, country) [101].

## Supplementary Materials

Supplementary materials can be found at http://www.mdpi.com/1422-0067/20/3/717/s1.

## Abbreviations

Anx	Annexin
BK	Big potassium
CRAC	Cholesterol recognition amino-acid consensus
ER	Endoplasmic reticulum
LDL	Low-density lipoprotein
LEL	Late endolysosome
LSD	Lysosomal Storage Disorder
MCS	Membrane contact site
mTOR	mechanistic Target of Rapamycin
NPC	Niemann–Pick type C
NPCD	Niemann–Pick type C disease
NSF	N-ethylmaleimide Sensitive Fusion
NTD	N-terminal domain
PI(3,5)P_2_	Phosphatidylinositol-3,5-bisphosphate
PI(4,5)P_2_	Phosphatidylinositol-4,5-bisphosphate
RCK	Regulation of conductance by potassium
RMS	Root mean square
SM	Sphingomyelin
SNARE	Soluble NSF protein attachment receptor
Sph	Sphingosine
SSD	Sterol-sensing domain
Stx	Syntaxin
TPC	Two-pore channel
TRPML	Transient receptor potential mucolipin
VAMP	Vesicle-associated membrane protein
