# Supplementary material for: Lipid–Protein Interactions in Niemann–Pick Type C Disease: Insights from Molecular Modeling
Source: Int J Mol Sci. 2019 Feb 7;20(3):717. doi: 10.3390/ijms20030717 (PMC6387118; doi:10.3390/ijms20030717)
Supplement: Supplementary file 1 [file ijms-20-00717-s001.zip › ijms-437724-supp figures.pdf]

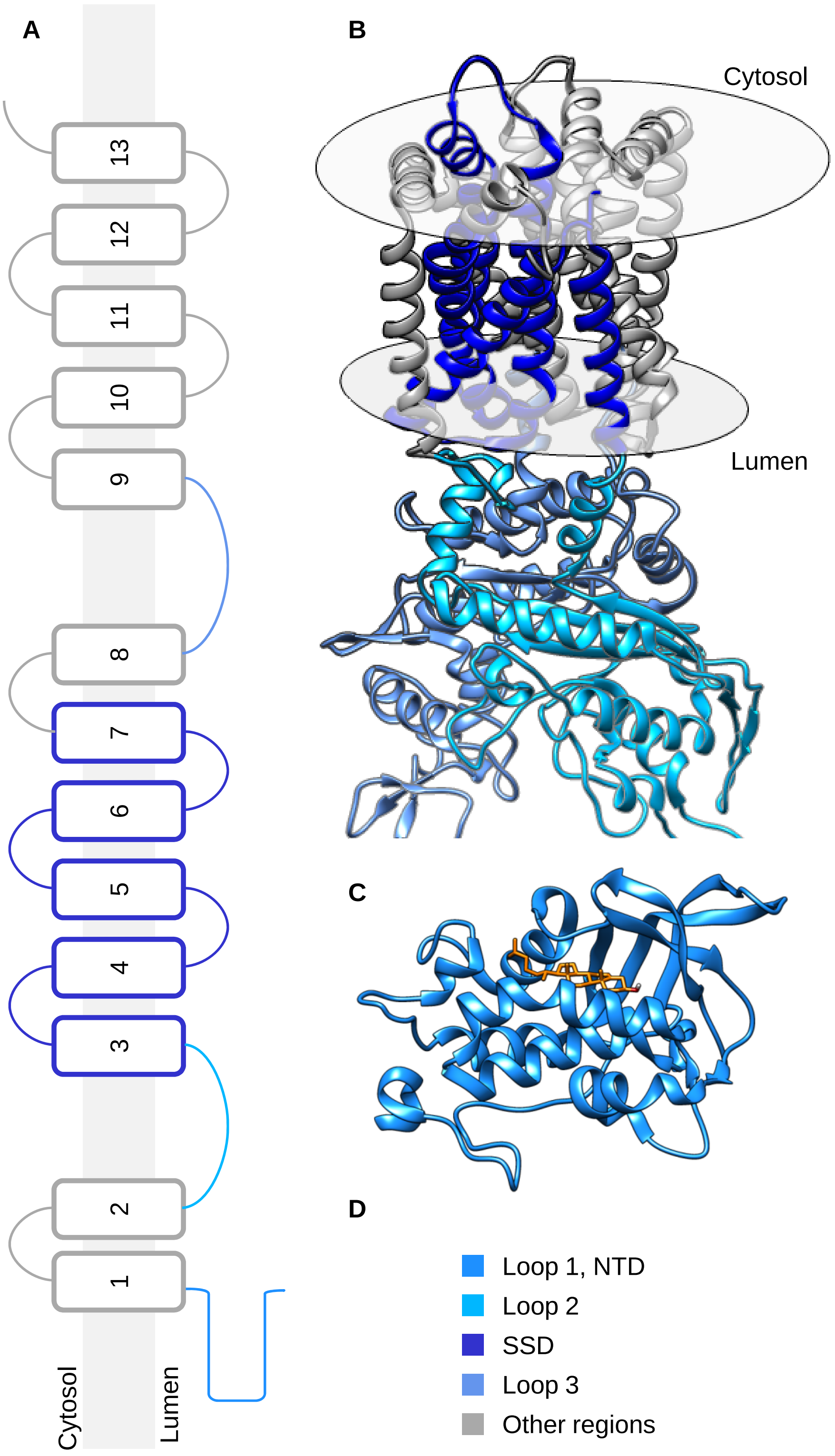

Figure S1 Structure of the NPC1 protein showing its different regions (**A**) Cartoon of full protein (**B**) Ribbon depiction of residues 334-1278 based on PDB 5U74 (**C**) Ribbon depiction of NTD based on PDB 3GKI (cholesterol orange) (**D**) Colour key.

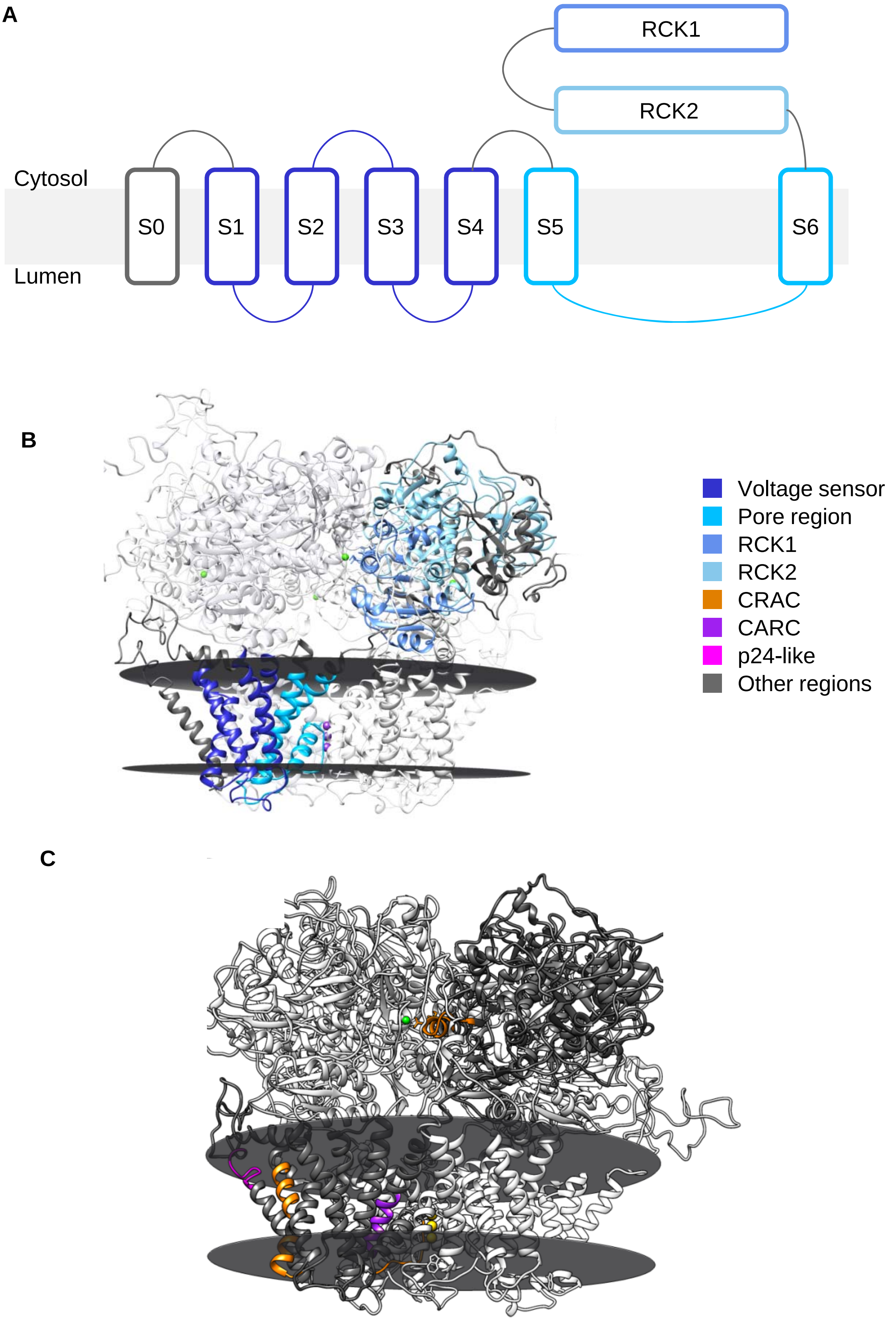

Figure S2 The BK channel A: functional domains in a cartoon representation of one of the 4 proteins that comprise the homotetramer. **B**: functional domains shown in one of the proteins from the model based on 5tj6. **C**: Putative lipid binding domains of interest. In **B** and **C** three of the proteins are shown in white for clarity

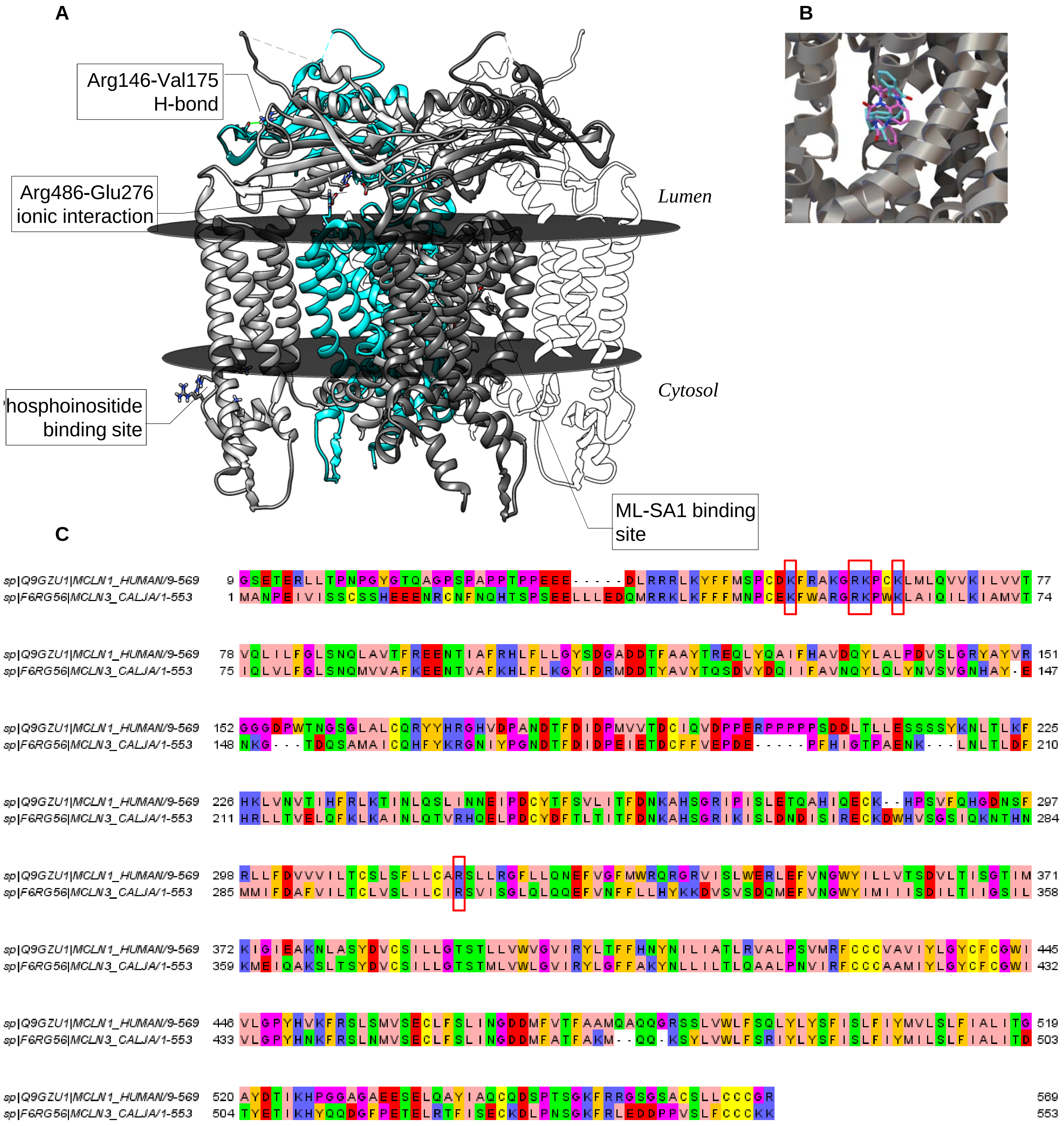

Figure S3 Possible SM binding sites on the TRPML1 channel A: SM may bind at one of 4 locations – two ligand binding sites and two sites of interaction between neighbouring protein chains. Chains are shown in different colours and the approximate location of the membrane is indicated with discs. **B**: Docking reproduces the binding mode of synthetic ligand ML-SA1 **C**: Alignment of human TRPML1 (PDB: 5WJ9) and marmoset TRPML3 (PDB: 5W3S) locates conserved residues (red boxes) of the phosphoinositide binding site.

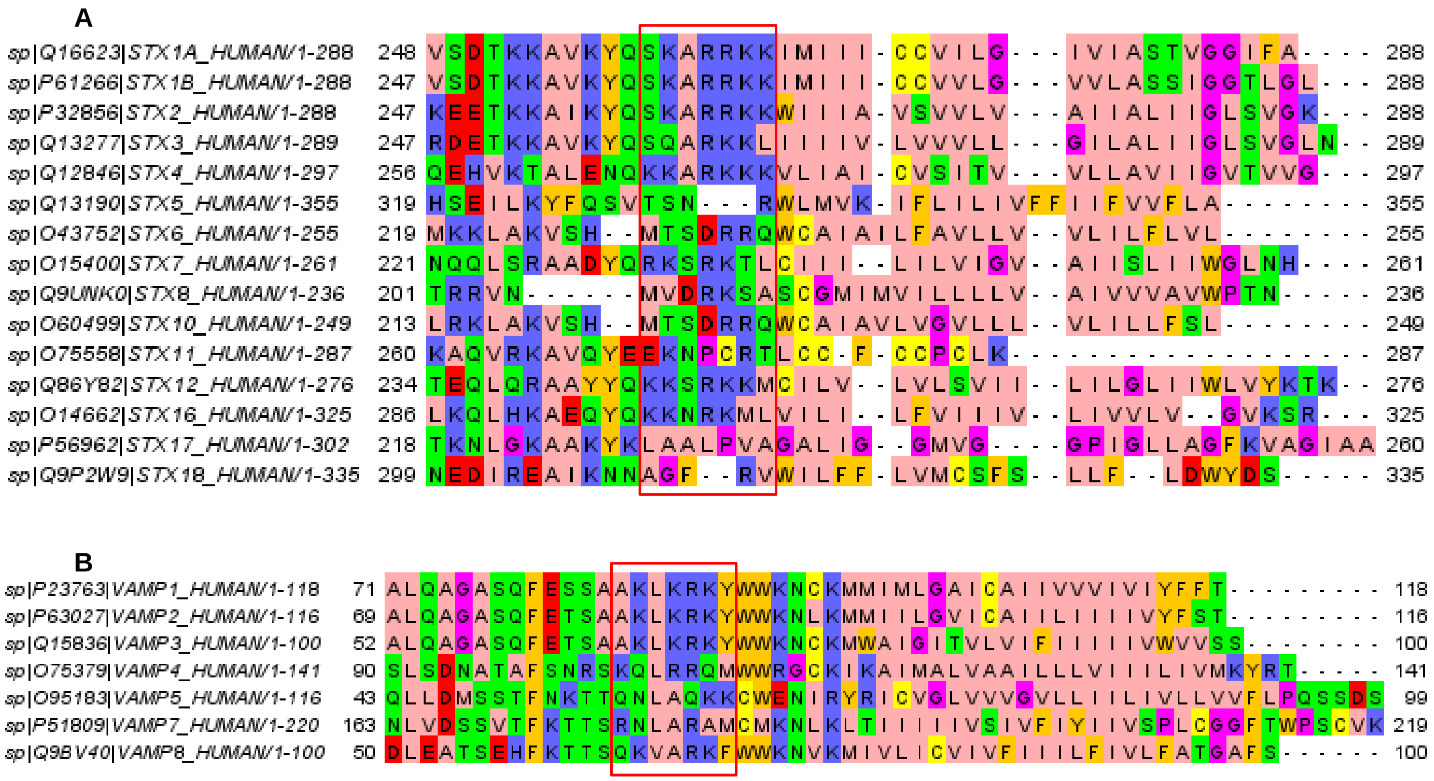

Figure S4 Multi-sequence alignment of human SNARE proteins. Both the syntaxin (**A**) and VAMP (**B**) families feature a polybasic section (red boxes) in the juxta-membrane region.

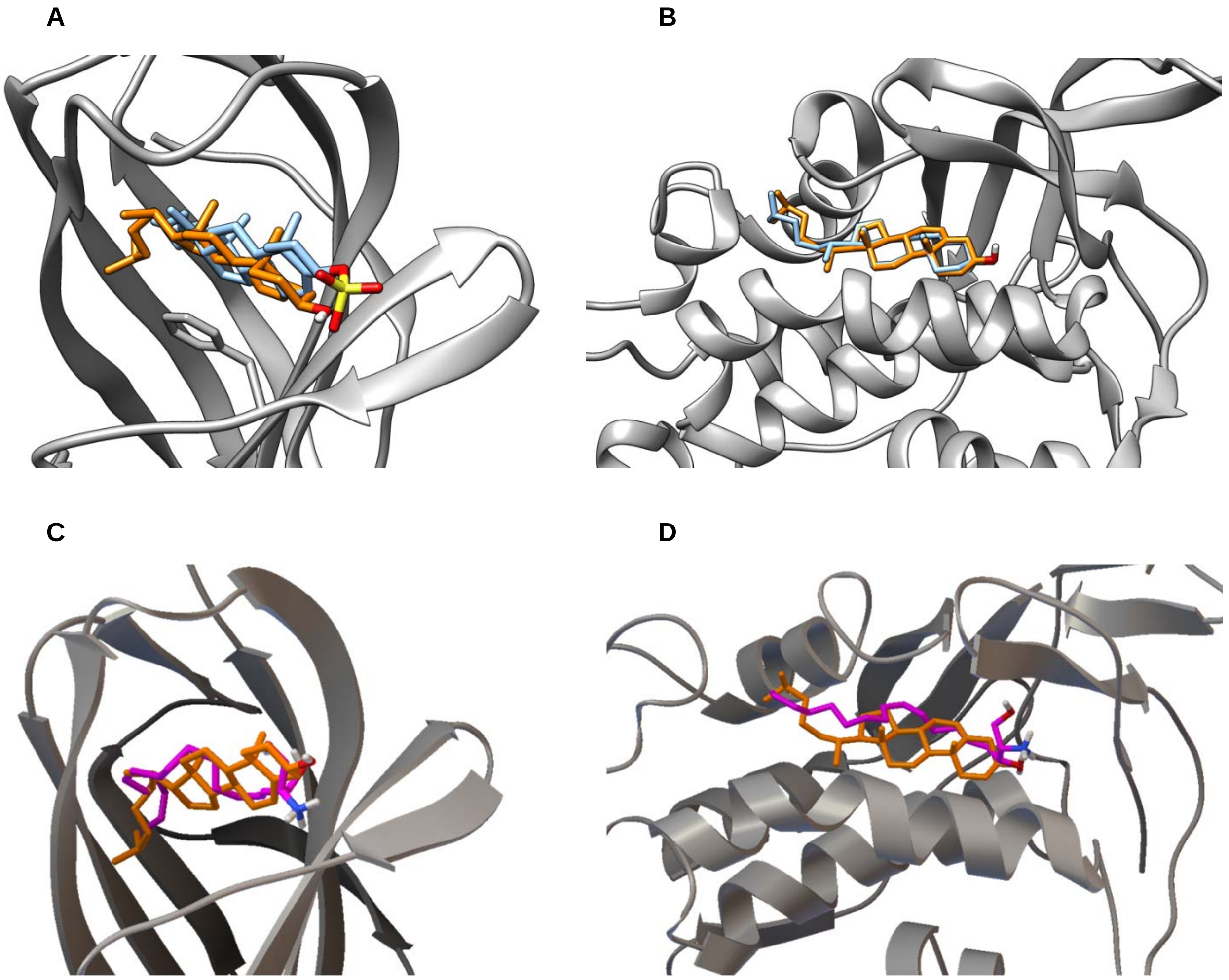

Figure S5 Cholesterol and sphingosine binding to NPC proteins A: Docking of cholesterol (orange) accurately reproduces the binding of cholesterol-O-sulfate (blue) to NPC2 in PDB 5KWY; both lipids interact with Phe85 via the smooth α face. **B:** Docking of cholesterol (orange) accurately reproduces the binding to NTD of NPC1 in PDB 3GKI. **C:** Cholesterol (orange) and sphingosine (purple) docked to NPC2 **D:** Cholesterol (orange) and sphingosine (purple) docked to the N-terminal domain of NPC1. The lipophilic and hydrophilic parts of cholesterol and sphingosine are positioned similarly in **C** and **D**.

**Protein**	**Structure**	**Model based on**	**Reso-lution (Å)**	**QMEAN-Brane score**	**Position in membrane**	**Refer-ence**
NPC1	5U74		3.335	-4.23	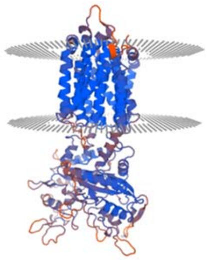	6
BK (membrane region)		5TJI (55.7% identical)	3.8	-5.85	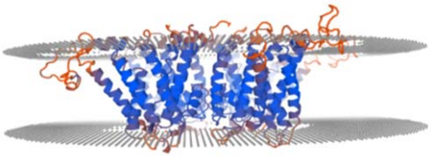	22,23
TRPML1	5WJ9		3.49	-5.08	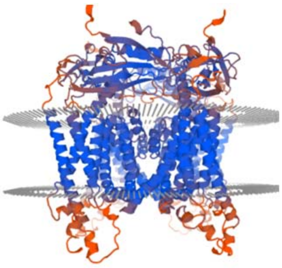	64
AnxA2	2HYW		2.1	0.48	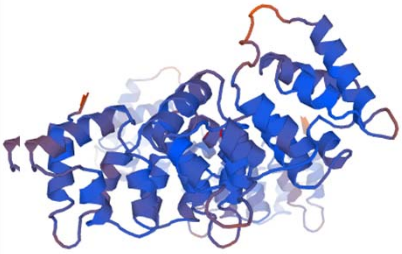	76
Stx7 VAMP8 bundle		3HD7 (Stx7 36.2% identical, VAMP8 35.6% identical)	3.4	-2.87	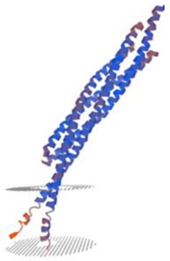	78

Figure S5: Protein structures and models used in this study The PDB IDs are either of the relevant protein or the protein used to build a model. QMEANBrane scores give a measure of the quality of structures/model; higher is better. QMEAN was used for soluble proteins. Cartoons are given by QMEAN and give an impression of local quality of the model from blue (good) to orange (bad)

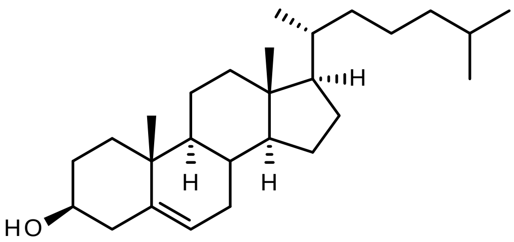

Cholesterol

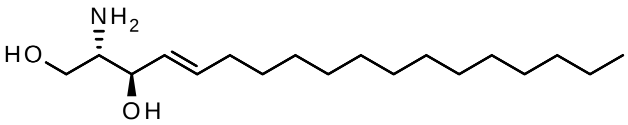

Sphingosine

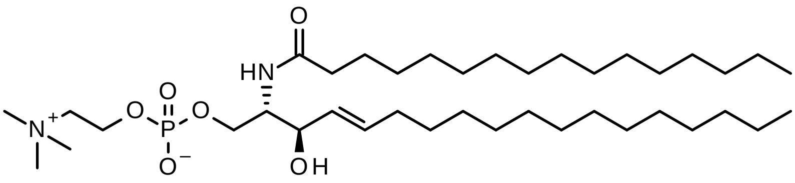

Sphingomyelin

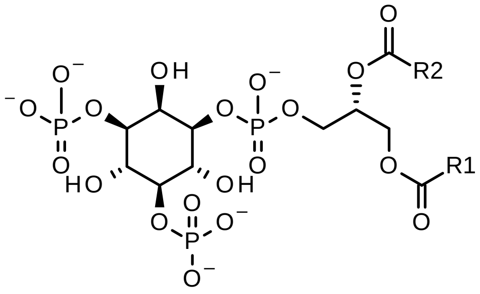

PI(3,5)P_2 head group
R1 and R2 are long hydrocarbon chains

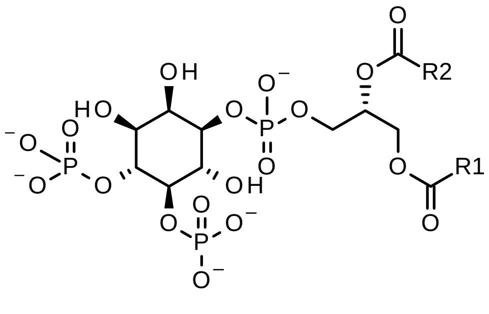

PI(4,5)P_2 head group
R1 and R2 are long hydrocarbon chains

Figure S6 Lipids studied in this work

**Protein, region**	**Grid centre**	**Grid dimensions (points)**	**Grid offests (points)**	**Residues with flexible sidechains**	**Lipid and (number of GA runs)**
NPC1,<br>SSD	Phe779,<br>Cβ	x=80<br>y=76<br>z=126	x=-7.583<br>y=5.5<br>z=7	none	Cholesterol (100)<br>Sphingosine (300)
BK,<br>CRAC2	Tyr263,<br>Cα	x=46<br>y=40<br>z=60	x=0<br>y=-2.5<br>z=3.75	Lys211<br>Asn265<br>Tyr263	Cholesterol (300)
TRPML1,<br>Juxta-membrane	Asp114 (chain A),<br>CZ	x=40<br>y=50<br>z=60	x=10<br>y=-3.75<br>z=0	Glu276 (chain A)<br>Arg486 (chain D)	Spingomyelin (300)
TRPML1,<br>agonist	Pro52,<br>Cγ	x=40<br>y=40<br>z=40	x=8.5<br>y=-6.5<br>z=0	Lys55<br>Arg61<br>Lys65<br>Arg318	PI(3,5)P_2,<br>PI(4,5)P_2,<br>Sphingomyelin (all 500)
AnxA2	Gly279,<br>Cα	x=60<br>y=40<br>z=60	x=0<br>y=-2.75<br>z=-2	Lys279<br>Lys281<br>Arg284	PI(4,5)P_2<br>(400)
Stx7/VAMP8,<br>juxta-membrane 1	Lys62 (VAMP8),<br>Cα	x=60<br>y=60<br>z=60	x=4<br>y=0<br>z=0	Lys66 (VAMP8)<br>Lys72 (VAMP8)<br>Arg235 (Stx7)	PI(3,5)P_2<br>(600)
Stx7/VAMP8,<br>juxta-membrane 2	Lys161 (Stx7),<br>NZ	x=54<br>y=58<br>z=50	x=-1.5<br>y=0<br>z=-1.75	Arg67 (VAMP8)<br>Lys233 (Stx7)	PI(3,5)P_2<br>(400)

Figure S7 Particulars of docking procedures In AutoDock the search space is centred on an atom and has a length in points (1 point = 0.375Å) in each of the three spatial dimensions. This space can be moved by the specified offest amounts. While AutoDock holds the protein backbone rigid, it does allow the sidechains of certain residues to be made flexible. The various descriptors of the ligand's relation to the protein (position, orientation, bond angles) and then altered and a certain amount of mixing between different states allowed; hence the procedure is termed a genetic algorithm (GA). For more details on the procedures used to perfrom docking and calculate the grids used, see references 99 and 100.
