# Supplementary material for: Lipid–Protein Interactions in Niemann–Pick Type C Disease: Insights from Molecular Modeling
Source: Int J Mol Sci. 2019 Feb 7;20(3):717. doi: 10.3390/ijms20030717 (PMC6387118; doi:10.3390/ijms20030717)
Supplement: Supplementary file 1 [file ijms-20-00717-s001.zip › ijms-437724-supp table.pdf]

Protein, region	Lipid	Cluster (members)[a]	Mean binding energy (kcal/mol)	Status	Position<br>Interactions (side-chain van der Waals unless stated)	Member shown in text
NPC1, SSD	Cholesterol	1 (74)	-9.50	Parallel, favoured	Luminal<br>Asp620 (side-chain, H-bond), Val 621, Val624, Val625, Tyr628, Leu665, Val668, Ile687, Glu688, Pro691	1 (-0.38)
		2 (4)	-9.42	Parallel, favoured	Luminal<br>Ser627 (side-chain, H-bond), Val 621, Val624, Val625, Ala629, Tyr628 Phe632, Ile687, Glu688, Pro691	
		4 (3)	-8.93	Anti-parallel, disfavoured	-	
		5 (7)	-8.51	Parallel, favoured	Cytosolic[b]<br>Ile654, Ile361Ala695, Leu663, Ser655, Cys778, Leu782, Leu785, Asp786 (side-chain, H-bond), Arg789	1 (-0.25)
		6 (10)	-8.25	Anti-parallel, disfavoured	-	
	Sphingosine	1 (115)	-5.67	Parallel, favoured	Luminal<br>Ser627 (backbone, H-bond), Asp620 (side-chain, H-bond), Val 621, Val625, Tyr628, Ile687, Glu688, Pro691, Phe1221	1 (-1.36)
		2 (47)	-5.53	Parallel, favoured	Luminal<br>Ser617 (backbone, H-bond), Asp620 (side-chain, H-bond), Val 624, Val668, Leu672, Val686, Ile687, Leu682, Glu688, Ile690, Pro691, Phe1221	
		3 (24)	-5.59	Parallel, favoured	Luminal<br>Ser617, Asp620 (side-chain, H-bond), Val621, Val 624, Tyr628, Leu665, Val668, Ala669, Glu688 (side-chain, H-bond), Pro691, Phe1221	
		4 (22)	-5.30	Parallel, favoured	Luminal<br>Asp620 (side-chain, H-bond), Val621, Val 624, Val625, Tyr628, Phe632, Leu665, Glu688, Pro691, Phe1221	
		5 (5)	-5.26	Parallel, favoured	Luminal<br>Asp620 (side-chain, H-bond), Val621, Val 624, Val625, Tyr628, Phe632, Ile687, Glu688, Pro691, Phe1221	
		6 (8)	-4.43	Parallel, favoured	Luminal<br>Asp620, Val 624, Tyr628, Leu665, Ile687, Glu688 (side-chain, H-bond), Pro691, Leu695, Phe1221	
		7 (5)	-3.99	Anti-parallel, disfavoured	-	
		8 (13)	-4.54	Parallel, favoured	Cytosolic[b]<br>Ile354, Ile361, Ser655, Ile662, Ile663, Leu665, Cys778, Leu785, Asp786, Arg789, Asp796 (side-chain and backbone, H-bonds)	3 (-0.76)
		9 (5)	-4.76	Perpendicular, Disfavoured	-	
		10 (10)	-4.34	Parallel, favoured	Luminal<br>Ser617 (side-chain and backbone, H-bonds), Asp620 (side-chain, H-bond), Val621, Val 624, Val625, Tyr628, Ile687	
		11 (8)	-4.11	Anti-parallel, disfavoured	-	
		12 (9)	-4.39	Parallel, favoured	Luminal<br>Ser617 (side-chain and backbone, H-bonds), Asp618, Val621, Val 624, Tyr628, Phe632, Leu684, Pro691	
		13 (8)	-4.38	Perpendicular, Disfavoured	-	
BK, CRAC2	Cholesterol	1 (109)	-6.26	Displaced parallel, disfavoured	-	
		2 (72)	-6.05	Parallel, favoured	Lys211 (side-chain, H-bond), Leu215, Val223, Leu226, Val255, Val262, Tyr263, Asn265 (side-chain, H-bond)	22 (-0.27)
		3 (56)	-6.05	Parallel, favoured	Leu215, Val223, Leu226, Val255, Val262 (side-chain, H-bond and van der Waals), Tyr263, Asn265 (side-chain, H-bond)	
		4 (6)	-5.49	Parallel, favoured	Lys211 (side-chain, H-bond), Leu215, Val223, Leu226, Val255, Val262, Tyr263 (side-chain, CH-π)	4 (+0.34)
		5 (3)	-5.57	Perpendicular, disfavoured	-	

**Protein, region**	**Lipid**	**Cluster (members)[a]**	**Mean binding energy (kcal/mol)**	**Status**	**Position Interactions (side-chain van der Waals unless stated)**	**Member shown in text**
BK, CRAC2	Cholesterol	8 (7)	-5.63	Displaced parallel, disfavoured	-	
		9 (5)	-4.68	Anti-parallel, disfavoured	-	
		10 (3)	-4.93	Displaced parallel, disfavoured	-	
		11 (17)	-5.14	Anti-parallel, disfavoured	-	
TRPML1, Juxta-membrane	SM head group	1 (2)	-3.40	Splayed Disfavoured	-	
		2 (2)	-3.04	Anti-parallel, disfavoured	-	
		3 (3)	-2.60	Splayed Disfavoured	-	
		4 (3)	-2.35	Anti-parallel, disfavoured	-	
		6 (2)	-2.53	Anti-parallel, disfavoured	-	
		7 (2)	-3.12	Splayed Disfavoured	-	
		8 (2)	-3.05	Perpendicular, disfavoured	-	
		9 (8)	-1.41	Anti-parallel, disfavoured	-	
		11 (5)	-2.76	Anti-parallel, disfavoured	-	
		12 (6)	-1.71	Parallel, favoured	Asp114 (side-chain, H-bond), Glu276 (side-chain, ionic), Arg486 (side chain, ionic)	1 (-1.63)
		14 (4)	-2.21	Anti-parallel, disfavoured	-	
		15 (3)	-3.02	Splayed Disfavoured	-	
		16 (2)	-2.71	Perpendicular, disfavoured	-	
		17 (3)	-1.45	Perpendicular, disfavoured	-	
		20 (3)	-1.41	Splayed Disfavoured	-	
		21 (7)	-1.73	Anti-parallel, disfavoured	-	
		22 (2)	-2.50	Splayed Disfavoured	-	
		23 (2)	-1.92	Anti-parallel, disfavoured	-	
		25 (2)	-2.37	Perpendicular, disfavoured	-	
		28 (4)	-1.84	Anti-parallel, disfavoured	-	
		29 (3)	-1.44	Perpendicular, disfavoured	-	
		31 (2)	-2.37	Perpendicular, disfavoured	-	

Protein, region	Lipid	Cluster (members)[a]	Mean binding energy (kcal/mol)	Status	Position<br>Interactions (side-chain van der Waals unless stated)	Member shown in text
TRPML1, agonist	$PI(3,5)P_2$ headgroup [c]	1 (27)	-17.40	Parallel, favoured	Lys55 (side-chain, ionic), Arg67 (side-chain, ionic), Lys65 (side-chain, ionic), Arg318 (side-chain, ionic)	
	$PI(4,5)P_2$ headgroup [c]	2 (22)	-19.11	Parallel, favoured	Lys55 (side-chain, ionic), Arg67 (side-chain, ionic), Lys65 (side-chain, ionic), Arg318 (side-chain, ionic)	
	SM headgroup [c]	8 (9)	-6.05	Parallel, favoured	Lys55 (side-chain, ionic), Arg67 (side-chain, H-bond), Lys65 (side-chain, ionic), Arg318 (side-chain, H-bond), Arg322 (side-chain, H-bond)	
AnxA2	$PI(4,5)P_2$ headgroup [d]	1 (1)	-7.82	Parallel, favoured	Lys279 (side-chain, ionic), Lys281 (side chain, ionic and H-bond), Arg284 (side-chain, ionic)	
		2 (4)	-7.07	Parallel, favoured	Lys279 (side-chain, ionic and H-bond), Lys281 (side chain, ionic and H-bond), Arg284 (side-chain, ionic)	
		3 (4)	-4.07	Parallel, favoured	Lys279 (side-chain, ionic and H-bond), Lys281 (side chain, ionic and H-bond; backbone, H-bond), Arg284 (side-chain, ionic)	
		7 (3)	-2.76	Parallel, favoured	Lys279 (side-chain, ionic), Lys281 (side chain, H-bond), Arg284 (side-chain, ionic)	2 (+0.18)
		9 (3)	-3.32	Parallel, favoured	Lys281 (side chain, H-bond;), Arg284 (side-chain, ionic)	
		10 (2)	-4.36	Splayed, disfavoured	-	
		13 (2)	-4.73	Perpendicular, disfavoured	-	
		15 (4)	-3.48	Splayed, disfavoured	-	
		20 (3)	-3.62	Parallel, favoured	Lys279 (side chain, H-bond) Lys281 (side chain, H-bond), Arg284 (side-chain, ionic)	
		21 (4)	-4.00	Parallel, favoured	Lys279 (side-chain, H-bond), Lys281 (side chain, ionic and H-bond), Arg284 (side-chain, ionic)	
		25 (2)	-3.40	Splayed, disfavoured	-	
		27 (4)	-3.58	Anti-parallel, disfavoured	-	
		38 (2)	-3.37	Perpendicular, disfavoured	-	
		40 (2)	-3.88	Perpendicular, disfavoured	-	
		43 (3)	-3.73	Perpendicular, disfavoured	-	
		44 (3)	-3.31	Perpendicular, disfavoured	-	
		46 (2)	-4.02	Anti-parallel, disfavoured	-	
		50 (3)	-3.55	Perpendicular, disfavoured	-	
AnxA2	SM headgroup	1 (7)	-3.09	Anti-parallel, disfavoured	-	
		2 (3)	-3.34	Anti-parallel, disfavoured	-	
		3 (3)	-3.63	Anti-parallel, disfavoured	-	
		5 (3)	-2.36	Anti-parallel, Disfavoured	-	
AnxA2	SM	6 (4)	-3.17	Anti-parallel,	-	

Protein, region	Lipid	Cluster (members)[a]	Mean binding energy (kcal/mol)	Status	Position<br>Interactions (side-chain van der Waals unless stated)	Member shown in text
				disfavoured		
		8 (2)	-3.39	Perpendicular, disfavoured	-	
		9 (3)	-2.55	Splayed, disfavoured	-	
		1 (7)	-3.09	Anti-parallel, disfavoured	-	
		2 (3)	-3.34	Anti-parallel, disfavoured	-	
		3 (3)	-3.63	Anti-parallel, disfavoured	-	
		5 (3)	-2.36	Splayed, disfavoured	-	
		6 (4)	-3.17	Anti-parallel, disfavoured	-	
		8 (2)	-3.39	Splayed, disfavoured	-	
		9 (3)	-2.55	Anti-parallel, disfavoured	-	
		10 (3)	-2.83	Anti-parallel, disfavoured	-	
Stx7/VAMP8, juxta-membrane 1	PI(3,5)P_2 headgroup	1 (10)	-10.56	Parallel, favoured	Lys68 (VAMP8, side-chain, electrostatic and H-bond), Lys72 (VAMP8, side-chain, electrostatic), Arg235 (Stx7, side-chain, electrostatic and H-bond)	2 (-2.15)
		2 (3)	-11.58	Parallel, favoured	Lys68 (VAMP8, side-chain, electrostatic), Lys72 (VAMP8, side-chain, electrostatic and H-bond), Arg235 (Stx7, side-chain, electrostatic and H-bond)	
		3 (5)	-9.99	Anti-parallel, disfavoured	-	
		4 (5)	-10.70	Parallel, favoured	Lys68 (VAMP8, side-chain, electrostatic), Lys72 (VAMP8, side-chain, electrostatic and H-bond), Arg235 (Stx7, side-chain, electrostatic)	
		5 (2)	-12.97	Anti-parallel, disfavoured	-	
		6 (2)	-13.46	Anti-parallel, disfavoured	-	
		7 (6)	-9.83	Anti-parallel, disfavoured	-	
		8 (6)	-10.60	Anti-parallel, disfavoured	-	
		9 (10)	-9.94	Anti-parallel, disfavoured	-	
		10 (7)	-10.50	Anti-parallel, disfavoured	-	
		11 (8)	-9.91	Anti-parallel, disfavoured	-	
		12 (4)	-10.10	Parallel, favoured	Lys68 (VAMP8, side-chain, electrostatic), Lys72 (VAMP8, side-chain, electrostatic and H-bond), Arg235 (Stx7, side-chain, electrostatic and H-bond)	
		14 (8)	-10.00	Perpendicular, disfavoured	-	
		15 (2)	-11.20	Parallel, favoured	Lys68 (VAMP8, side-chain, electrostatic), Lys72 (VAMP8, side-chain, electrostatic), Arg235 (Stx7, side-chain, electrostatic and H-bond)	
		17 (6)	-9.97	Perpendicular, Disfavoured	-	

Protein, region	Lipid	Cluster (members)[a]	Mean binding energy (kcal/mol)	Status	Position<br>Interactions (side-chain van der Waals unless stated)	Member shown in text
Stx7/VAMP8, juxta-membrane 1	PI(3,5)P_2 headgroup	18 (6)	-8.82	Anti-parallel, disfavoured	-	
		19 (6)	-11.93	Parallel, favoured	Lys68 (VAMP8, side-chain, electrostatic), Lys72 (VAMP8, side-chain, electrostatic), Arg235 (Stx7, side-chain, electrostatic)	
		20 (3)	-10.36	Parallel, favoured	Lys68 (VAMP8, side-chain, electrostatic), Lys72 (VAMP8, side-chain, electrostatic), Arg235 (Stx7, side-chain, electrostatic)	
		22 (4)	-9.91	Parallel, favoured	Lys68 (VAMP8, side-chain, electrostatic and H-bond), Lys72 (VAMP8, side-chain, electrostatic), Arg235 (Stx7, side-chain, electrostatic and H-bond)	
		23 (3)	-10.91	Perpendicular, disfavoured	-	
		24 (6)	-9.48	Parallel, favoured	Lys68 (VAMP8, side-chain, electrostatic), Lys72 (VAMP8, side-chain, electrostatic), Arg235 (Stx7, side-chain, electrostatic and H-bond)	
		25 (7)	-8.68	Parallel, favoured	Lys68 (VAMP8, side-chain, electrostatic), Lys72 (VAMP8, side-chain, electrostatic and H-bond), Arg235 (Stx7, side-chain, electrostatic and H-bond)	
		26 (5)	-9.97	Parallel, favoured	Lys72 (VAMP8, side-chain, electrostatic), Arg235 (Stx7, side-chain, electrostatic)	
		28 (2)	-11.04	Perpendicular, disfavoured	-	
		30 (9)	-9.21	Perpendicular, disfavoured	-	
		31 (4)	-9.16	Perpendicular, disfavoured	-	
		32 (5)	-9.20	Parallel, favoured	Lys68 (VAMP8, side-chain, electrostatic), Lys72 (VAMP8, side-chain, electrostatic), Arg235 (Stx7, side-chain, electrostatic)	
Stx7/VAMP8, juxta-membrane 2[e]	PI(3,5)P_2 headgroup	1 (8)	-3.37	Perpendicular, disfavoured	-	
		2 (2)	-4.75	Parallel, favoured	Arg67 (VAMP8, side-chain, electrostatic), Lys233 (Stx7, side-chain, H-bond)	
		3 (4)	-2.75	Parallel, favoured	Arg67 (VAMP8, side-chain, electrostatic), Tyr230 (Stx7, side-chain, H-bond), Lys233 (Stx7, side-chain, electrostatic and H-bond)	1 (-2.50)
		4 (18)	-2.20	Anitparallel, disfavoured	-	
		5 (12)	-1.92	Parallel, favoured	Lys233 (Stx7, side-chain, electrostatic)	
		6 (4)	-2.76	Anitparallel, disfavoured	-	
		7 (8)	-2.23	Anitparallel, disfavoured	-	
		8 (6)	-2.45	Perpendicular, disfavoured	-	
		9 (8)	-2.69	Perpendicular, disfavoured	-	
		10 (5)	-3.41	Perpendicular, disfavoured	-	
		11 (4)	-3.30	Perpendicular, disfavoured	-	
		12 (3)	-2.99	Parallel, favoured	Lys233 (Stx7, side-chain, electrostatic and H-bond)	
		13 (12)	-2.65	Perpendicular, disfavoured	-	
		14 (6)	-2.87	Parallel, favoured	Lys233 (Stx7, side-chain, electrostatic)	

Protein, region	Lipid	Cluster (members)[a]	Mean binding energy (kcal/mol)	Status	Position<br>Interactions (side-chain van der Waals unless stated)	Member shown in text
		17 (3)	-3.68	Parallel, favoured	Lys233 (Stx7, side-chain, electrostatic)	
		19 (4)	-3.41	Anitparallel, disfavoured	-	
		20 (10)	-1.62	Anitparallel, disfavoured	-	
		21 (6)	-1.97	Perpendicular, disfavoured	-	
		22 (6)	-2.49	Parallel, favoured	Arg73 (VAMP8, side-chain, electrostatic), Lys233 (Stx7, side-chain, electrostatic)	
		23 (2)	-2.46	Perpendicular, disfavoured	-	
		24 (4)	-2.28	Parallel, favoured	Asn73 (VAMP8, side-chain, H-bond), Lys233 (Stx7, side-chain, electrostatic and H-bond)	
		25 (3)	-1.95	Anitparallel, disfavoured	-	
		26 (16)	-2.00	Parallel, favoured	Asn73 (VAMP8, side-chain, H-bond), Lys233 (Stx7, side-chain, electrostatic)	
		27 (10)	-2.16	Perpendicular, disfavoured	-	
		28 (2)	-3.45	Perpendicular, Disfavoured	-	
		29 (12)	-2.54	Perpendicular, Disfavoured	-	
		30 (4)	-2.03	Perpendicular, disfavoured	-	

Table S1 Details of AutoDock output AutoDock clusters binding poses by RMSD (cut-off > 2.0 Å) and orders clusters by binding energy of most favourable member. For each binding experiment the clusters are given with the number of members of each in parentheses. The mean binding energy is given; AutoDock's margin for error is ± 1.5 kcal/mol so clusters with mean energies within this margin are energetically indistinguishable. The status of each cluster (assessed as described in main text) is given. The interacting residues for each cluster are listed along with the position in the LEL membrane. The binding pose from each cluster chosen as representative and shown in the main text is indicated; the difference from the mean binding energy of the cluster (kcal/mol) is given in parenthesis – a negative value indicates more favourable and a positive value less favourable.

Notes: [a]Docking experiments generally produce a long 'tail' of small clusters with less favourable binding energies. These are omitted as are clusters with only 1 member. [b]The smaller size of the clusters alligned to the cytosolic face relative to those alligned to the luminal face is likely a consequce of the larger size of the luminal binding pocket. When cholesterol is positioned randomly at the start of the calculation run it is more likely to be close to the luminal pocket. The energetic favourability of binding poses aligned to the cytosolic face was confirmed by independent docking experiments using ROSIE-Rosetta software (rosie.rosettacommons.org/ligand_docking, data not shown). [c]Data given for the most energetically favourable and biologically plausible cluster only. [d]Increasing the RSMD cut-off to 4.0Å dramatically reduced the number of clusters but clustered some antiparallel binding poses together with parallel ones (data not shown). [e]RSMD cut-off of 2.5Å was used.
